# The endocannabinoid system in canine Steroid-Responsive Meningitis-Arteritis and Intraspinal Spirocercosis

**DOI:** 10.1371/journal.pone.0187197

**Published:** 2018-02-06

**Authors:** Jessica Freundt-Revilla, Franciska Heinrich, Alexander Zoerner, Felix Gesell, Martin Beyerbach, Merav Shamir, Anna Oevermann, Wolfgang Baumgärtner, Andrea Tipold

**Affiliations:** 1 Department of Small Animal Medicine and Surgery, University of Veterinary Medicine Hannover, Hannover, Germany; 2 Center for Systems Neuroscience, Hannover, Germany; 3 Department of Pathology, University of Veterinary Medicine Hannover, Hannover, Germany; 4 Institute for Clinical Pharmacology, Hannover Medical School, Hannover, Germany; 5 Institute for Biometry, Epidemiology, and Information Processing, University of Veterinary Medicine Hannover, Hannover, Germany; 6 Koret School of Veterinary Medicine, The Hebrew University of Jerusalem, Jerusalem, Israel; 7 Department Clinical Research and Veterinary Public Health, Vetsuisse Faculty, University of Bern, Bern, Switzerland; University of Pisa, ITALY

## Abstract

Endocannabinoids (ECs) are involved in immunomodulation, neuroprotection and control of inflammation in the central nervous system (CNS). Activation of cannabinoid type 2 receptors (CB2) is known to diminish the release of pro-inflammatory factors and enhance the secretion of anti-inflammatory cytokines. Furthermore, the endocannabinoid 2-arachidonoyl glycerol (2-AG) has been proved to induce the migration of eosinophils in a CB2 receptor-dependent manner in peripheral blood and activate neutrophils independent of CB activation in humans. The aim of the current study was to investigate the influence of the endocannabinoid system in two different CNS inflammatory diseases of the dog, i.e. Steroid-Responsive Meningitis-Arteritis (SRMA) and Intraspinal Spirocercosis (IS). The two main endocannabinoids, anandamide (AEA) and 2-AG, were quantified by mass spectrometry in CSF and serum samples of dogs affected with Steroid- Responsive Meningitis-Arteritis in the acute phase (SRMA A), SRMA under treatment with prednisolone (SRMA Tr), intraspinal Spirocercosis and healthy dogs. Moreover, expression of the CB2 receptor was evaluated in inflammatory lesions of SRMA and IS and compared to healthy controls using immunohistochemistry (IHC). Dogs with SRMA A showed significantly higher concentrations of total AG and AEA in serum in comparison to healthy controls and in CSF compared to SRMA Tr (p<0.05). Furthermore, dogs with IS displayed the highest ECs concentrations in CSF, being significantly higher than in CSF samples of dogs with SRMA A (p<0.05). CSF samples that demonstrated an eosinophilic pleocytosis had the highest levels of ECs, exceeding those with neutrophilic pleocytosis, suggesting that ECs have a major effect on migration of eosinophils in the CSF. Furthermore, CB2 receptor expression was found in glial cells in the spinal cord of healthy dogs, whereas in dogs with SRMA and IS, CB2 was strongly expressed not only in glial cells but also on the cellular surface of infiltrating leukocytes (i.e. neutrophils, eosinophils, lymphocytes, plasma cells, and macrophages) at lesion sites. The present study revealed an upregulated endocannabinoid system in dogs with inflammatory CNS diseases, highlighting the endocannabinoid system as a potential target for treatment of inflammatory CNS diseases.

## Introduction

In the last decades an increasing interest emerged in the use of derivatives of the plant *Cannabis sativa* and *Cannabis indica* commonly known as Marijuana to treat a variety of disorders both in humans and animals. One of the main reasons for such an effort is that numerous cannabinoids have potential medicinal effects lacking the psychoactive effects of some phytocannabinoids like ∆9–tetrahydrocannabinol (THC) [1, 2]. These compounds interact mainly with two receptors, cannabinoid receptor type 1 (CB1) and cannabinoid receptor type 2 (CB2), which are expressed in several tissues in mammals [3] and together with their endogenous ligands (endocannabinoids) and the enzymes responsible for their synthesis and degradation constitute the endocannabinoid system [4, 5].

Endocannabinoids (ECs) are endogenous lipid transmitters that mimic the action of THC by binding and activating the cannabinoid receptors [6]. The most bioactive ECs and the best studied ones are Anandamide (AEA) and 2-arachidonoylglycerol (2-AG) [4, 6, 7]. Anandamide was firstly identified in the porcine brain as an endogenous ligand of the CB1 receptor [8], and shows low affinity to CB2 receptors [9]. 2-AG was isolated from canine intestine, being the first endocannabinoid isolated from peripheral tissue [10]. However, 2-AG was also isolated from the rat brain [11]. Endocannabinoids bind to local receptors only and are immediately inactivated under physiological conditions [12].

CB1 receptors are expressed mostly on neurons [13] and mediate the inhibition of neurotransmitter release [3], while CB2 receptors are highly expressed on immune cells [14] modulating cytokine release [3], decreasing antigen presentation [15] and modulating cell migration [1]. As a result, many of the medicinal properties of cannabinoid compounds have been attributed to the CB2 receptor, especially those related to immune system modulation [1]. In dogs, CB2 receptors have been identified in Although CB2 receptors are not strongly expressed in the brain under normal conditions, in neuroinflammatory diseases an up-regulation occurs in microglial and glial cells [16] allowing the endocannabinoid system to function as an immune modulator in both the peripheral immune system as well as the central nervous system (CNS) [17].

Increasing evidence supports the immunomodulatory roles of 2-AG and AEA [18]. Moreover, exogenous application of 2-AG and AEA has shown to exert anti-inflammatory effects by decreasing the production of inflammatory mediators [19]. The anti-inflammatory and neuroprotective effects of endocannabinoids and cannabinoids have been shown in several experimental models [20–26], where activation of the endocannabinoid system has been linked to decreased inflammatory cell recruitment and enhanced anti-inflammatory cytokine production [18]. Therefore, the endocannabinoid system could be established as a promising target for the treatment of inflammatory disorders.

The aim of the current study was to investigate in a comparative way the influence of the endocannabinoid system in two different inflammatory CNS diseases of the dog, *i*.*e*. SRMA and IS. SRMA is a systemic immune disorder characterized by inflammatory lesions of vessels, particularly in the cervical leptomeninges [27]. *Spirocerca Lupi* is a nematode parasite of dogs and other carnivores which affects mainly the esophagus and aorta [28] and aberrant migration to the spinal cord parenchyma has been previously reported [29, 30]. The following hypotheses should be proven: (i) endocannabinoids can be measured in cerebrospinal fluid (CSF) in inflammatory diseases; (ii) the endocannabinoid system depends on the etiology of the inflammatory CNS disease and might influence the differential cell count of CSF; (iii) CB2 receptors are up-regulated in inflammatory lesions of SRMA and IS. For this purpose, the endocannabinoids, AEA and 2-AG were measured in CSF and serum samples by mass spectrometry and CB2 receptor expression was evaluated in inflammatory lesions in the spinal cord of dogs with SRMA and IS using routine immunohistochemistry.

## Materials and methods

### Serum and cerebrospinal fluid samples

A total of 41 cerebrospinal fluid (CSF) samples and 36 serum samples were retrospectively analyzed. From them, 27 paired serum and CSF samples were collected between July 2008 and December 2012 from client-owned dogs diagnosed with SRMA at the Department of Small Animal Medicine and Surgery, University of Veterinary Medicine Hannover, Germany. Additionally, 3 serum and 7 CSF samples from seven dogs affected with IS presented to the Koret School of Veterinary Medicine Teaching hospital, The Hebrew University of Jerusalem, Israel. Finally, 6 paired CSF and serum samples from healthy Beagles from the Department of Small Animal Medicine and Surgery, University of Veterinary Medicine Hannover, Germany, were used as controls. This study was conducted in accordance with the ethical guidelines of the University of Veterinary Medicine Hannover and was approved by the authorities of Lower Saxony (Animal experiment number 33.9-42502-05-12A214). CSF samples were collected from the cerebellomedullary cistern or lumbar subarachnoidal space of the dogs under general anaesthesia, and serum samples were obtained by puncture of the cephalic or saphenous peripheral vein. Routine CSF analysis was performed immediately after collection and the remaining CSF and serum samples were stored at -20°C until further analysis.

SRMA in the acute stage (SRMA A) was defined by occurrence of cervical rigidity and pain, fever and polymorphonuclear pleocytosis in the CSF [31]. Other possible causes of neck pain, elevated temperature and/or pleocytosis in CSF were ruled out. Dogs in SRMA A group were not pre-treated with glucocorticosteroids prior to CSF puncture. On the other hand, dogs in the SRMA treatment (Tr) group were under long-term treatment with prednisolone as previously described [32] and did not show any clinical signs at the time of sampling. Furthermore, they displayed routine CSF parameters in physiological ranges (cell count: 0–3 cells/μl; glucose 60–80% of the blood glucose concentration; protein < 25 mg/dl) [33].

Inclusion criteria for IS required dogs to live in areas endemic for *Spirocerca Lupi* and the combination of the following characteristic clinical and pathological findings: acute onset of non-symmetrical hind limb paralysis, radiographic or endoscopic evidences of esophageal granuloma and eosinophilic pleocytosis. Favorable response to treatment supported the diagnosis in dogs that survived and detection of the nematode within the spinal cord parenchyma confirmed the diagnosis in dogs that were euthanized due to lack of improvement.

Serum and CSF samples were used for determination of both endocannabinoids (AEA and 2-AG) by stable-isotope dilution liquid chromatography combined with tandem mass spectrometry (LC-MS/MS) [34, 35].

### Animals and tissue samples

A total of 8 dogs with SRMA, 2 dogs with confirmed IS, and 5 healthy controls were included in the immunohistochemical evaluation. The healthy control group consisted of 5 Beagles without clinical or pathological evidence of neurologic or infectious diseases. Tissue samples were obtained following routine necropsy and were included in previous studies [36, 37] conducted in accordance with the German Animal Welfare Act with the law of animal welfare, Germany (permission number: 33.9-42502-05-13A346), and following the ethical guidelines of the University of Veterinary Medicine Hannover. No animals were euthanized for this particular study; samples obtained and previously used in other studies were taken. The study wasapproved and followed the guidelines of the PhD commission of the University ofVeterinary Medicine Hannover, the institutional ethics committee.

Spinal cord tissue samples from six of the 8 dogs with SRMA originated from the Department of Clinical Research and Veterinary Public Health, Vetsuisse University of Bern Switzerland, and two were obtained from the Department of Pathology, University of Veterinary Medicine Hannover, Germany. Tissue samples from dogs with confirmed intraspinal migration of *Spirocerca Lupi* were sent from Korea School of Veterinary Medicine, The Hebrew University of Jerusalem, Israel.

Sections of the cervical, thoracic, and lumbar spinal cord were collected during necropsy and fixed in 10% formalin for at least 24 hours. After fixation, tissue samples were embedded in paraffin, cut at serial sections (3 μm), mounted onto coated slides (SuperFrost-Plus® slides; Menzel Gläser, Braunschweig, Germany), and stained with hematoxylin and eosin (HE). Subsequently, a complete histological examination in order to detect histopathological lesions and immunohistochemical staining to evaluate CB2 expression were performed. Given the fact that CB2 receptors are strongly expressed in immune tissues and leukocyte subpopulations [14], spleen and liver tissue of a healthy dog served as positive control and was processed equally.

Details of breed, sex, age, and morphological changes of the lesions and diagnosis from all dogs are summarized in Table 1.

**Table 1:** Sex, age, anatomic localization of the lesions, morphological diagnosis in dogs suffering SRMA, IS and control dogs.

Case	Breed	Sex	Age	Spinal cord section analyzed	Morphological changes in spinal cord				Diagnosis
					Vasculitis	Meningitis	Hemorrhage	Myelomalacia	
1	Beagle	M	6m	Cervical	-	-	-	-	Healthy (Control dog)
2	Beagle	M	6m	Cervical	-	-	-	-	Healthy (Control dog)
3	Beagle	M	6m	Cervical	-	-	-	-	Healthy (Control dog)
4	Beagle	F	6m	Cervical	-	-	-	-	Healthy (Control dog)
5	Beagle	F	6m	Cervical	-	-	-	-	Healthy (Control dog)
6	Great Dane	M	5y, 10m	Cervical, thoracic, lumbar	++	++	-	+	SRMA
7	Mixed breed	M	8m	Cervical	+++	+++	+++	+++	SRMA
8	Tibetan Terrier	M	1y	Cervical	+++	+++	++	+++	SRMA
9	n-a	F	7m	Cervical, thoracic, lumbar	++	++	-	-	SRMA
10	Newfoundland	F	n-a	Thoracic	++	++	-	-	SRMA
11	Bernese Mountain Dog	F	n-a	Cervical, thoracic, lumbar	+++	+++	++	-	SRMA
12	Labrador Retriever mix	M	6y	Cervical	+++	++	+	-	SRMA
13	German Shepherd	M	10m	Cervical, thoracic, lumbar	+++	+++	-	-	SRMA
14	Golden Retriever	F	4y	Thoracic, lumbar	+	++	+++	+++	Intraspinal Spirocercosis
15	Mixed breed	F	n-a	Thoracic	+	+	+++	+++	Intraspinal Spirocercosis

CNS: Central Nervous System; SRMA: steroid-responsive meningitis-arteritis; IS: intraspinal spirocercosis; y: years; m: months; n-a: not available; M: male; F: female; absent: -; mild: +; moderate: ++; severe: +++.

### Liquid chromatography combined with tandem mass spectrometry (LC-MS/MS)

The quantification of AEA and 2-AG in CSF and serum samples was performed by stable-isotope dilution liquid chromatography combined with tandem mass spectrometry (LC-MS/MS) as previously described [34, 35]. Briefly, after thawing on ice, Hydroxypropyl-β-cyclodextrine (10% w/v) was added to CSF samples prior to liquid-liquid extraction to improve extraction efficacy. This step was not required for serum samples. Additionally, 500 μl of the internal standards d_4_-AEA (Nr. 10011178, Cayman chemical, Michigan, USA) and d_5_-2-AG (Nr. 36162, Cayman chemical, Michigan, USA) were added to each sample, to a final concentration of 1.1 nM and 1.0 nM, respectively. This step was performed on ice. Subsequently, 500 μl Toluene (Nr. 244511, Sigma-Aldrich®, Steinheim, Germany) were added to each sample and homogenization was performed by shaking twice during 20 seconds at 5000 rpm at 4°C in a PreCellys tissue homogenizer. After centrifugation (5 min, 4500 xg, 4°C) the organic phase was separated and evaporated to dryness by a gentle stream of nitrogen for approximately 50 min. Finally, 50 μl eluent (25% water and 75% methanol) were added to the residues for liquid chromatographic analysis and 25 μl of the solution was injected into the Waters ACQUITY/XEVO TQ-MS LC-MS/MS system (Waters, Milford, MA, USA). Chromatographic separation took place on a Waters ACQUITY BEH C18 reversed phase column (100 mm × 2.1 mm ID, 1.7 μM particle size) [35]. The following transitions were monitored: m/z348→m/z 62 (AEA), m/z352→m/z66 (d4-AEA), m/z 379→m/z 287 (2-AG), and m/z 384→m/z287 (d5-2-AG). Due to the quick isomerization of 2-AG to its less biologically active form 1-arachidonoylglycerol (1-AG) [38], quantification of 2-AG is difficult to accomplish [39]. The available CSF and serum samples in the current study contained a large portion of 1-AG as compared to 2-AG. Therefore, 2-AG was calculated and referred to as total AG concentration using the sum of the 2-AG and 1-AG peak areas in the acquired chromatograms as previously described [34].

### Immunohistochemistry

Immunohistochemistry (IHC) was performed using the avidin-biotin-peroxidase complex (ABC) method as previously described [36, 40]. Briefly, formalin-fixed paraffin-embedded sections of the selected tissue samples were dewaxed and rehydrated in a descending alcohol dilution series. Inhibition of endogenous peroxidases was performed in 85% ethanol with 0.5% H_2_O_2_ for 30 minutes at room temperature. Slides were pre-treated with sodium-citrate buffer (pH 6.0–6. 5) in the microwave at 800w for 20 minutes for antigenic retrieval. In order to prevent non-specific binding of the antibodies, all slides were incubated with normal goat serum (1:5 in phosphate-buffered saline [PBS]) for 20 min at room temperature. Following blocking, slides of interest and positive controls were incubated in a moist chamber with a polyclonal anti-CNR2/CB2 antibody (IHC-plus^TM^ LS-A34, LSBio, LifeSpan BioSciences, Inc., Seattle, WA, USA; dilution 1:100 in PBS containing 1% bovine serum albumin [BSA]) overnight at 4°C. The primary antibody used cross-reacts with human, monkey and canine CB2, as the canine CB2 sequence shares between 76 and 82% homology with rat, mouse and human CB2 [41]. As negative control, the primary antibody was substituted by rabbit serum (R4505; Sigma Aldrich, Taufkirchen, Germany; dilution 1:3000 in PBS with 1% BSA) and incubated for the same time period. Subsequently, a secondary biotin-labelled goat-anti-rabbit antibody (Vector Laboratories, Burlingame, CA, USA; dilution 1:200 in PBS) was incubated on all slides for 45 minutes at room temperature, followed by incubation with ABC (VECTASTAIN-ABC Kit Standard, PK 6100, Vector Laboratories, Burlinghame, California, USA). Finally, slides were incubated with the chromogen diaminobezidine-tetrahydrochloride (0.05% solution, DAB, Sigma Aldrich, Taufkirchen, Germany) and 0.03% H_2_O_2_ for 5 minutes at room temperature followed by a slight counterstaining with Mayer's hemalaun, dehydration in an ascending series of alcohol, cleaning in acetic acid-n-butylester (EBE®, Roth, Karlsruhe, Germany), and mounting using Roti®-Histokit (Roth, Karlsruhe, Germany). Sections of tissue samples were independently examined via light microscopy (BX51, Olympus Optical CO., Tokyo, Japan) and representative images were acquired by use of photodocumentation software (DP72, Olympus Optical CO., Tokyo, Japan).

### Statistical analysis

Statistical analysis to quantify differences between the levels of AEA and total AG among the groups was performed using the SAS® software Version SAS 9.3 (SAS Inst. Inc. Cary, North Carolina, USA). Normal distribution of the data was found after logarithmic transformation. For descriptive statistics, measures of location and statistical dispersion were depicted as median and range due lognormal distribution of data. Group differences of endocannabinoid concentrations were tested using One-way-analysis of variance for independent samples followed by the Least Significant Difference (LSD) test, a test controlling Type I comparison wise error rate. Differences between the groups were considered significant when the corresponding p-value was lesser than 0.05. Graphics from the statistical data obtained were performed using GraphPad® Software (GraphPad Prism, version 5, La Jolla, California, USA).

## Results

AEA and total AG were analyzed in 36 serum and 41 CSF samples. The distribution of the samples analyzed for AEA and total AG are summarized in Table 2.

**Table 2:** Number of CSF and serum samples analysed for AEA and total AG.

**Groups**	**AEA/Total AG**	
	CSF	Serum
**SRMA A**	9	9
**SRMA Tr**	19	18
**Healthy**	6	6
**Spirocercosis**	7	3
**Total**	41	36

AEA: Anandamide; Total AG: 1-AG (1-arachidonoylglycerol) + 2-AG (2-arachidonoylglycerol); CSF: cerebrospinal fluid; SRMA A: steroid-responsive meningitis-arteritis in acute stage; SRMA Tr: SRMA dogs under treatment.

The group of dogs with SRMA included 6 females (1 neutered) and 15 males (1 neutered), ranged between 6 months and 27 months of age (median 17 months). Furthermore, this group included 12 different breeds mostly Boxers (31.81%) and Bernese Mountain dogs (18.18%). In some dogs with SRMA samples were taken twice, in the acute phase (SRMA A) and during treatment (SRMA Tr). All CSF samples in SRMA A and SRMA Tr and groups were obtained from the cerebellomedullary cistern. Dogs in the SRMA A group displayed a neutrophilic pleocytosis (Median: 619 cells/3μl; range: 7–2016 cells/3μl).

Seven dogs with IS were included in the study. These dogs presented with acute onset of either monoparesis (n = 2), paraparesis (n = 4) or paraplegia (n = 1) of the pelvic limbs, hyperesthesia in the lumbar (n = 2), lumbosacral (n = 1), and both cervical and lumbosacral (n = 1) regions, proprioceptive deficits of the pelvic limbs (n = 2), fecal and urinary incontinence (n = 2), reduced withdrawal reflex in the pelvic limbs (n = 1) and/or ataxia of all four limbs with hypometric gait of the thoracic limbs (n = 1). Complete blood count, chemistry profile and spinal radiographs were all normal except for elevated creatinine in one dog and mild leukocytosis in two dogs.

Further evaluations included serology for Toxoplasma and Neospora infection which was negative (n = 5). CSF samples from the IS group were taken from either the cerebellomedullary cistern or lumbar subarachnoidal space and showed mixed neutrophilic pleocytosis with moderate to severe eosinophilia.

Clinical diagnosis was further supported by improvement under treatment or at postmortem examination with identification of *Spirocerca Lupi* nematodes. Four dogs improved clinically under treatment, two deteriorated progressively and the owners opted for euthanasia. At postmortem examination a *Spirocerca Lupi* nematode was identified in the caudal lumbar segments of the spinal cord and a granuloma was found in the esophagus. Outcome of 1 dog is missing.

The healthy group was represented by 6 neutered-male Beagles, 9 months old, clinical and neurological examinations revealed no pathological findings. CSF was obtained from the cerebellomedullary cistern in these dogs.

### AEA and total AG in CSF and serum

Levels of AEA in CSF were found in the picomolar range (pM), in serum in the nanomolar range (nM). Concentrations of total AG in serum and CSF were expressed in the nanomolar range (nM).

### Levels of AEA in CSF and serum

The type 3 test of fixed effects denies the hypothesis of equal means of AEA for all group levels in CSF (F = 9.82, p<0.0001) and serum (F = 4.62, p = 0.0085).

The highest values of AEA in CSF were found in dogs with Intraspinal Spirocercosis and differed significantly with all other groups SRMA A (p = 0.0084), SRMA Tr (p<0.0001) and healthy dogs (p = 0.0001). Levels of AEA in CSF were significantly increased in SRMA A compared to SRMA Tr (p = 0.0482). Furthermore, healthy controls showed significantly lower concentrations of AEA in serum in comparison to dogs with Intraspinal Spirocercosis (p = 0.0032), SRMA A (p = 0.0111), and SRMA Tr (p = 0.0029).

**Fig 1:**
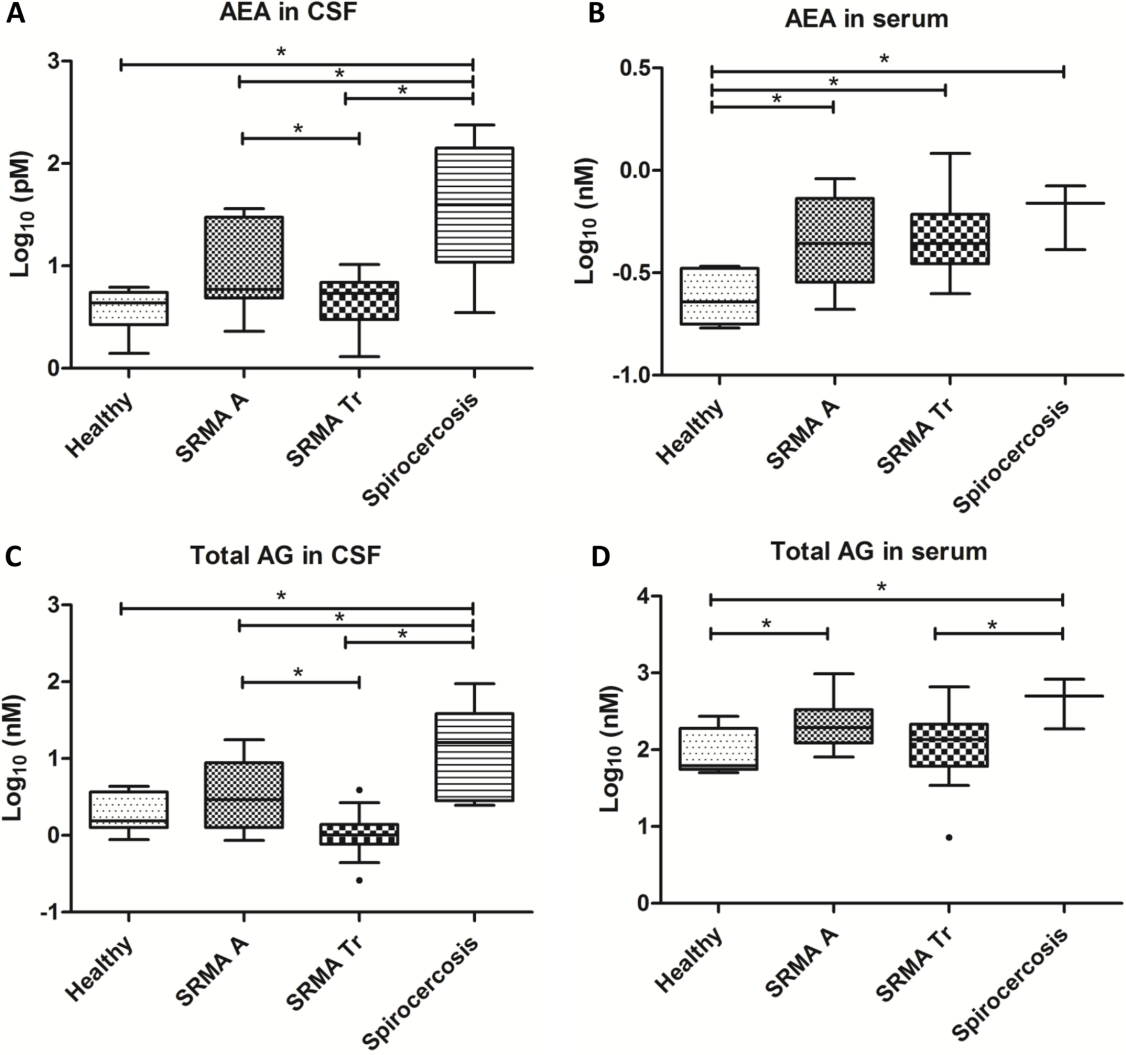
Concentrations of AEA and Total AG in CSF and serum samples, Log to base 10. Boxes contain values from 1^st^ to 3^rd^ quartile, central lines inside the boxes represent median values, and endpoints of vertical lines represent minimum and maximum values, dot (●) represent outliners. Asterisks (*) indicate statistically significant differences (p<0.05). AEA: Anandamide; Total AG: 1-AG (1-arachidonoylglycerol) + 2-AG (2-arachidonoylglycerol); CSF: cerebrospinal fluid; SRMA A: steroid-responsive meningitis-arteritis in acute stage; SRMA Tr: SRMA dogs under treatment; pM: picomolar; nM: nanomolar.

Medians and ranges of AEA concentrations in CSF and serum are summarized in Table 3. Significant differences among the groups are displayed in Fig 1.

**Table 3:** Levels of AEA and Total AG in CSF and serum samples analyzed: medians and ranges (minimum-maximum values).

		AEA		Total AG	
Groups		CSF (pM)	Serum (nM)	CSF (nM)	Serum (nM)
**Healthy**	Median	4.40	0.23	1.55	61.84
	Range	(1.40–6.20)	(0.17–0.34)	(0.88–4.35)	(50.17–272.90)
**SRMA A**	Median	5.90	0.44	2.91	193.92
	Range	(2.30–36.30)	(0.21–0.91)	(0.86–17.59)	(80.39–976.15)
**SRMA Tr**	Median	5.40	0.43	1.01	149.05
	Range	(1.30–10.30)	(0.25–1.21)	(0.26–3.91)	(34.20–659.17)
**Spirocercosis**	Median	39.40	0.69	16.13	500.10
	Range	(3.50–237.90)	(0.41–0.84)	(2.46–94.38)	(186.35–827.85)

AEA: Anandamide; Total AG: 1-AG (1-arachidonoylglycerol) + 2-AG (2-arachidonoylglycerol); CSF: cerebrospinal fluid; SRMA A: steroid-responsive meningitis-arteritis in acute stage; SRMA Tr: SRMA dogs under treatment; pM: picomolar; nM: nanomolar

### Levels of total AG in CSF and serum

The type 3 test of fixed effects denies the hypothesis of equal means of total AG for all group levels in CSF (F = 14.44; p<0.0001) and serum (F = 3.37; p = 0.0305).

Levels of Total AG in CSF were significantly increased in Intraspinal Spirocercosis compared to SRMA A (p = 0.0072), SRMA Tr (p< 0.0001) and healthy dogs (p = 0.0005). Furthermore, statistically significant differences were found between SRMA A and SRMA Tr, (p = 0.0016). Moreover, dogs suffering from SRMA in the acute phase and those with Intraspinal Spirocercosis showed significantly higher concentrations of total AG than healthy controls in serum (p = 0.0423 and p = 0.0080, respectively).

Medians and ranges of total AG concentrations in CSF and serum are summarized in Table 3. Significant differences among the groups are displayed in Fig 1.

### Spatiotemporal localization of CB2 receptors in healthy dogs

**Fig 2:**
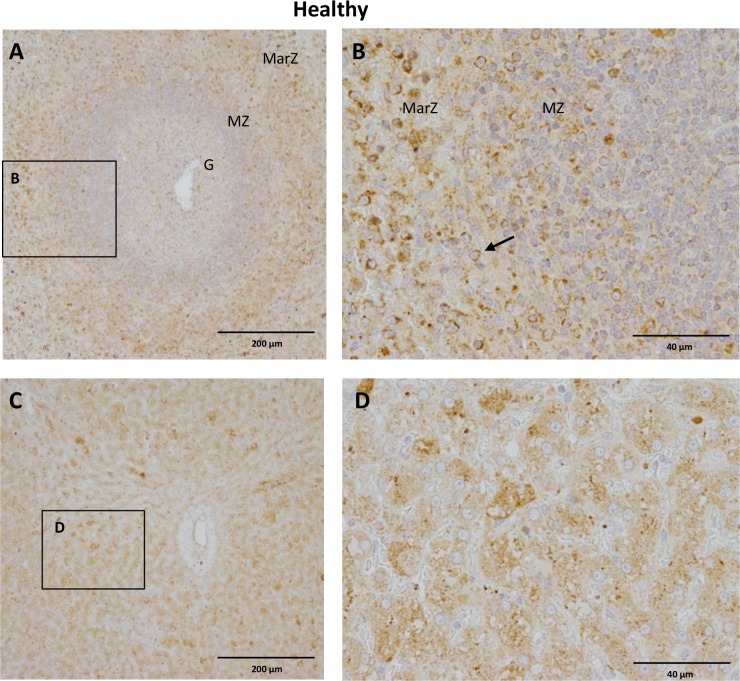
CB2 immunoreaction in spleen and liver of a healthy control dog. A) CB2 is expressed in numerous leukocytes in the marginal zone (macrophages, dendritic cells, transit B and T cells) and mantel zone (T cells) of splenic follicle (white pulp) whereas few positive cells are seen in the germinal center (B Cells). B) Insert/Close up of the marginal and mantel zone of splenic follicle showing numerous CB2 positive leukocytes (arrow). C) CB2 is abundantly expressed in hepatocytes and Kupffer cells as well as slightly in endothelial cells while tunica media and adventitia remain negative. D) Insert depicting CB2 positive Hepatocytes and Kupffer cells. G: Germinal center; MZ: mantel zone; MarZ: marginal zone. IHC was performed using the avidin-biotin-peroxidase complex (ABC) method.

Cannabinoid receptor type 2 immunoreaction was detected in spleen and liver samples of healthy dogs serving as positive controls. Moderate to strong immunoreaction was detected in numerous cells of the red pulp, as well as in the periarteriolar lymphoid sheath (PALS), marginal and mantel zone of splenic follicles (white pulp), while only few positive cells were found in the germinal center of splenic follicles (Fig 2A and 2B). Hepatocytes as well as Kupffer cells showed strong CB2 immunoreaction, while endothelial cells showed slight immunoreaction and smooth muscle cells of the tunica media were negative for staining with CB2 antibody (Fig 2C and 2D).

**Fig 3:**
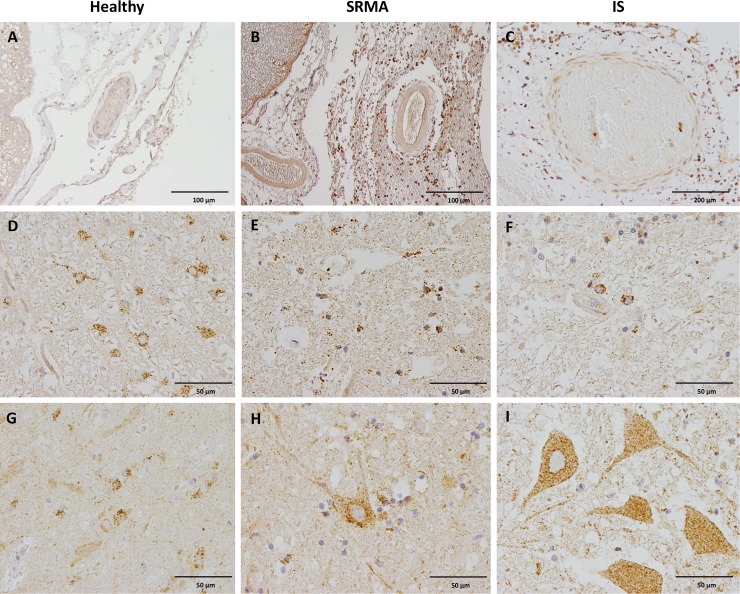
CB2 immunoreaction in spinal cord sections of dogs with SRMA, IS and healthy controls. A) Detail of blood vessel and meninges of a healthy dog, notice lack of immunoreaction in the adventitia, endothelial cells, flat mesothelial-like arachnoid membrane cells and pia matter cells. B) Blood vessel of a dog with SRMA characterized by strong perivascular and subarachnoidal CB2 positive inflammatory infiltrates and slight to moderate CB2 immunoreaction within the smooth muscle cells of the tunica media. C) Blood vessel of a dog with IS, slight CB2 immunoreactivity is seen in the tunica media of the vessel whereas perivascular inflammatory infiltrates show strong immunoreaction. D) Detail of glial cells in the white mater of a healthy dog with moderate to strong intracytoplasmic CB2 immunoreaction. E) Strong CB2 immunostaining is shown in round glial cells within the white matter of a dog with SRMA. F) Strong CB2 immunoreaction in astrocytes within the white matter of a dog with IS. G) Moderate to strong immunolabeling of astrocytes and slight immunolabeling of neurons with CB2 antibody within the grey matter of a healthy dog. H) Moderate CB2 immunostaining is shown in the cytoplasm of a neuron and adjacent glial cells within the grey matter of a dog with SRMA. I) Strong spotted CB2 immunoreaction in the cytoplasm of neurons within the grey matter of a dog with IS. IHC was performed using the avidin-biotin-peroxidase complex (ABC) method.

In spinal cord sections of healthy control dogs, CB2 was moderately to strongly expressed in numerous small vesicles in the cell membrane by approximately 90% of glial cells which were homogeneously distributed throughout the grey and white matter (Fig 3D and 3G). Moreover, slight to moderate immunoreaction was present in the cytoplasma of neurons in the ventral and dorsal horns. No CB2 immunoreaction was detectable in the tunica media and interna of blood vessels. Similarly, endothelial cells, flat mesothelial-like arachnoid membrane cells and pia mater cells lacked CB2 expression in the healthy state (Fig 3A).

### Spatiotemporal localization of CB2 receptors in SRMA lesions

**Fig 4:**
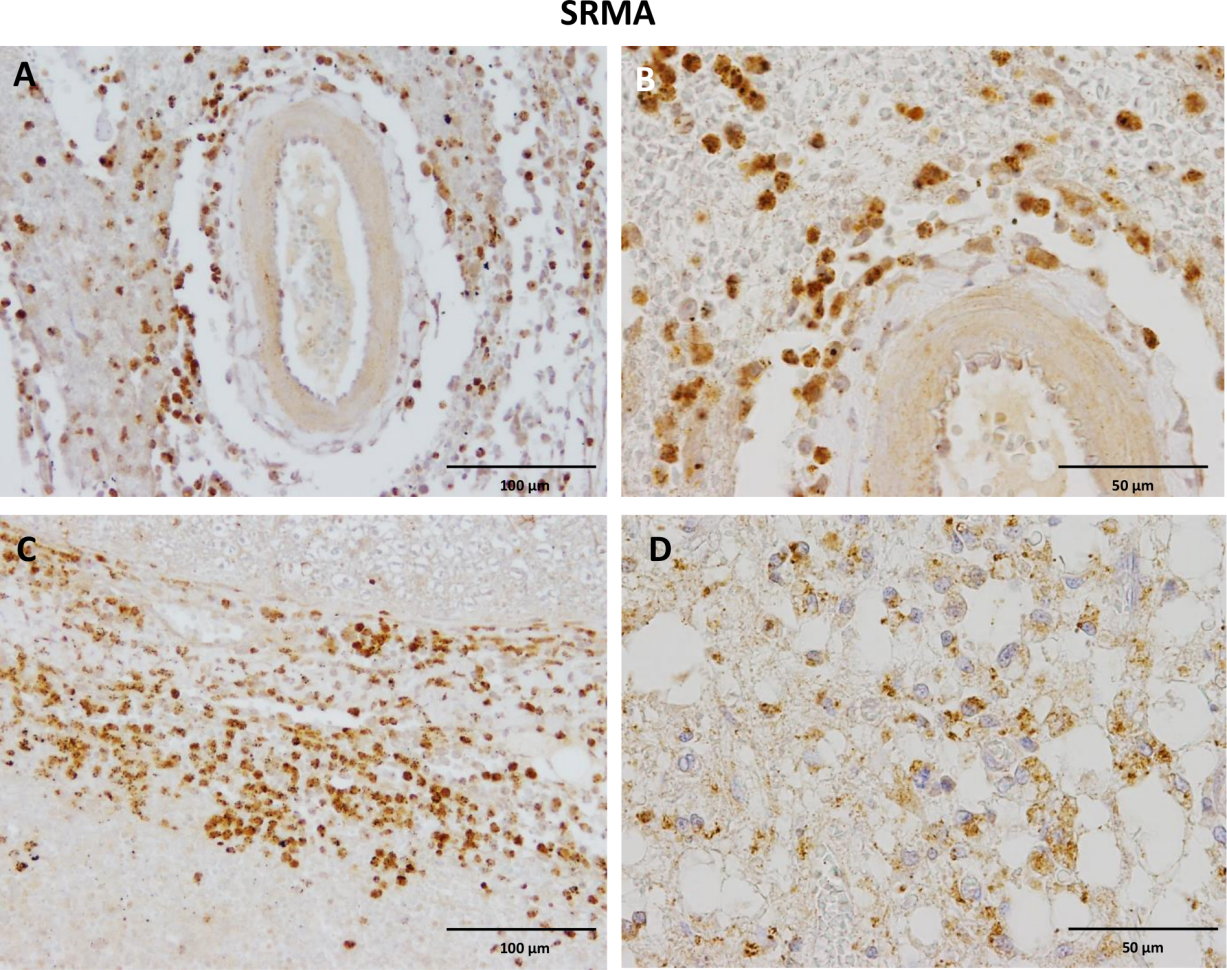
Immunohistochemistry of anti-CB2 antibody in spinal cord SRMA lesions. A) Strong CB2 positive perivascular inflammatory infiltrates surrounding meningeal blood vessels. B) The CB2 positive leukocyte population mainly consists of lymphocytes, plasma cells and fewer macrophages. C) Severe diffuse infiltration of the dura mater with CB2 positive inflammatory cells. D) Vacuolization of white matter areas with concomitant CB2 positive glial cells and leukocytes. IHC was performed using the avidin-biotin-peroxidase complex (ABC) method.

In cervical, thoracic, and lumbar spinal cord sections of dogs with SRMA, a constant finding in all examined dogs, was strong CB2 expression on infiltrating leukocytes (*i*.*e*. lymphocytes, plasma cells, neutrophils, and macrophages). In most dogs, inflammatory infiltrates were found within the adventitia of vessels, perivascular, subdural, and subarachnoidal, but in few dogs inflammatory cells were found within the white and grey matter (Fig 4A, 4B, 4C and 4D). Most vessels of the SRMA dogs showed thickened walls with a subendothelial spindle cell and collagen intimal proliferation which remained devoid of CB2 immunoreactivity. Endothelial cells lacked CB2 immunoreaction except rare scattered CB2 positive single cells. Slight to moderate CB2 immunoreactivity was observed in smooth muscle cells in the tunica media of medium to large caliber arteries (Fig 4A and 4B). Additionally, different CB2 immunoreaction patterns were identified in the spinal cords: on the one side, moderate, diffuse, homogeneous staining of the white matter (not shown), on the other side, negative staining of white matter areas with contrasting strong CB2-positive glial cells (Fig 3E). Moreover, the morphology of glial cells that labelled with CB2 antibody varied between sections. In some sections, mainly ramified glial cells (50–90%), presumably astrocytes, demonstrated a moderate to strong CB2-immunoreaction both in the grey and white matter which was comparable to healthy controls (Fig 3E and 3H). In contrast, in other sections, CB2 was strongly expressed by small round glial cells (10–50%), presumably oligodendrocytes, within the white and grey matter. Generally, neurons of the dorsal and ventral horns within the grey matter showed inhomogeneous spotted slight to strong cytoplasmic CB2 immunoreactivity (Fig 3H). Notably, also moderate to strong CB2-positive arachnoid mesothelial-like cells as well as pia mater cells near the lesions were present (Fig 3B).

### Spatiotemporal localization of CB2 receptors in IS lesions

**Fig 5:**
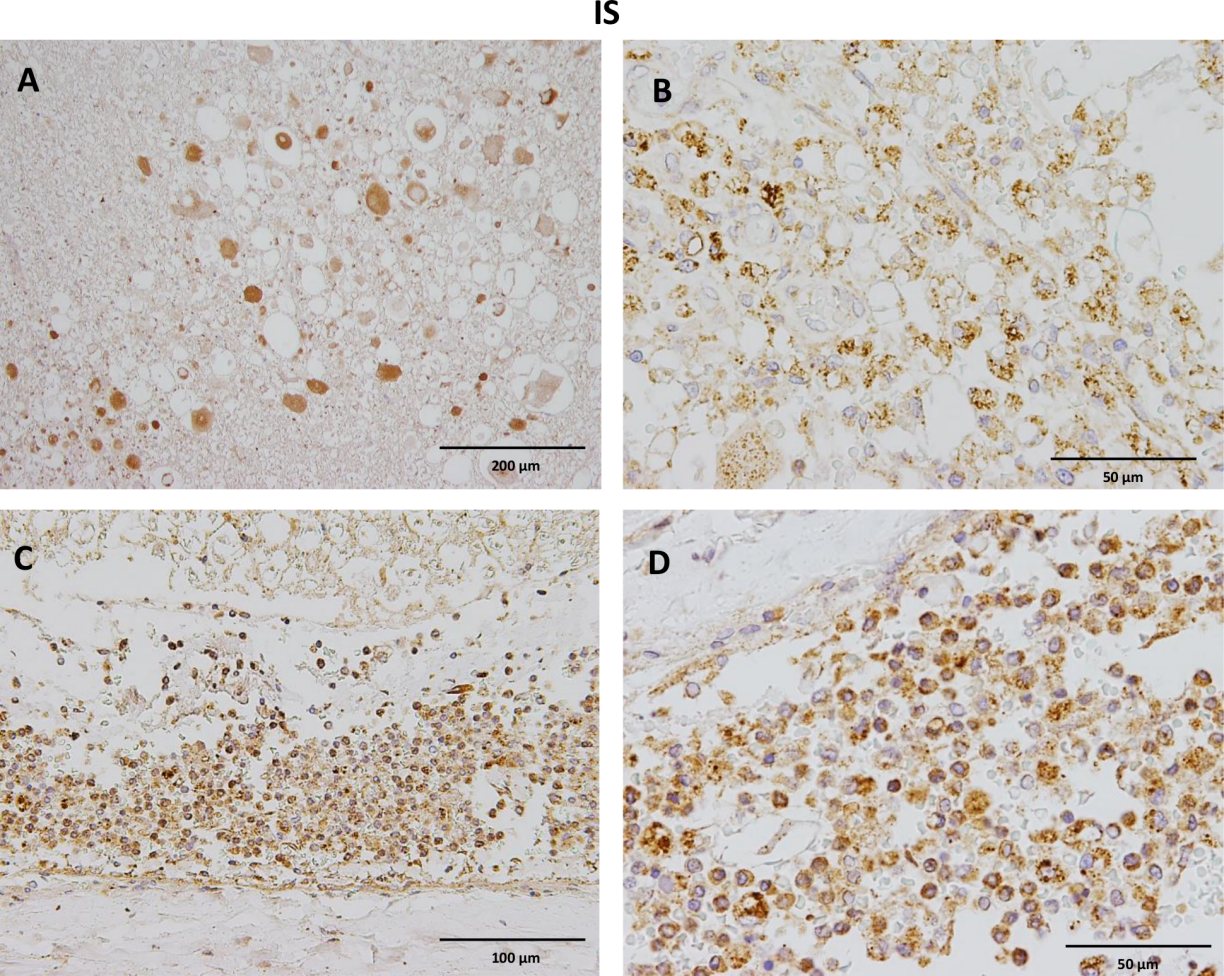
Immunohistochemistry of anti-CB2 antibody in spinal cord lesions of dogs with intraspinal spirocercosis. A) Notice moderate to strong CB2 immunostaining of swollen axons (spheroids) within the white matter. B) Prominent intracytoplasmic staining of large foamy macrophages (gitter cells) with CB2 antibody. C) Severe diffuse infiltration of the subarachnoidal space with strongly CB2 labeled inflammatory cells. D) The CB2 positive leukocyte population mainly consists of macrophages and fewer lymphocytes and plasma cells. IHC was performed using the avidin-biotin-peroxidase complex (ABC) method.

On thoracic spinal cord sections of dogs with IS, strong CB2 expressing leucocytes, predominantly lymphocytes, plasma cells and macrophages but also eosinophils and neutrophils were found adjacent to the parasite or the parasite tracts, accompanied by severe, multifocal to coalescing necrosis and hemorrhage but also within the subarachnoidal space (Fig 5B, 5C and 5D). Scattered glial cells stained moderately to strongly positive near the lesions and obtained a round-shaped morphology (Fig 3F), while glial cells in the white and grey matter distant from the lesion expressed low CB2 immunoreaction. Interestingly, moderate to severe axonal degeneration was found in the white matter with swollen CB2 positive axons (spheroids) and marked dilation of myelin sheets (Fig 5A). Neurons showed inhomogeneous moderate to strong cytoplasmic immunoreactivity within the dorsal and ventral horns (Fig 3I). Endothelial cells within the white and grey matter, but also in the subarachnoidal space showed slight cytoplasmic immunoreaction (Fig 3C).

**Fig 6:**
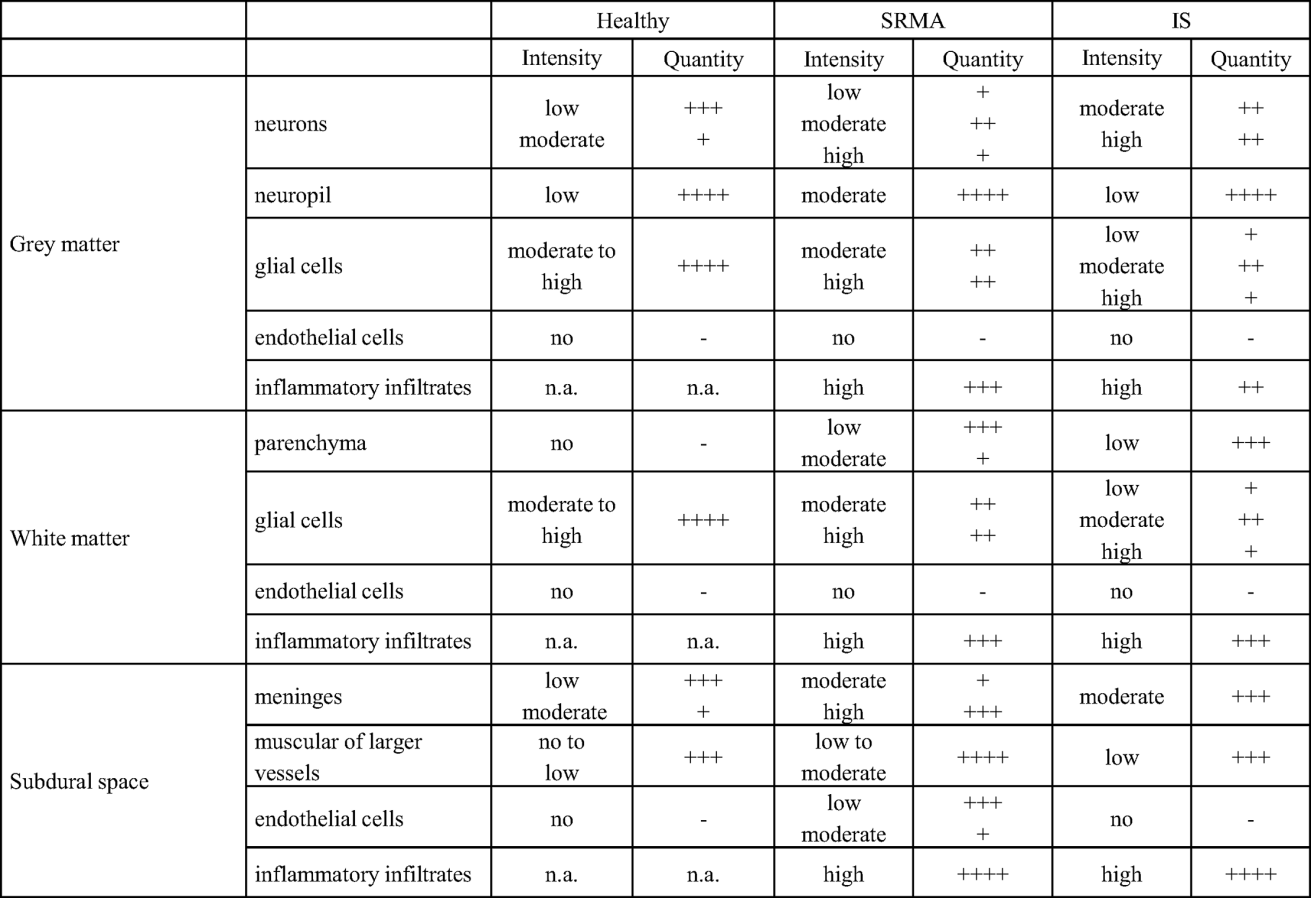
CB2 immunoreaction in the spinal cord of dogs with SRMA, IS and healthy controls. SRMA: steroid-responsive meningitis-arteritis; IS: intraspinal spirocercosis. Intensity: no, low, moderate, high. Quantity: -, no; +, < 10%; ++, 10–50%; +++, 50–90%, and ++++, >90% immunopositive cells; n.a. not applicable.

A semi quantitative analysis of the CB2 immunoreaction in the spinal cord of dogs with SRMA, IS and healthy controls at the cellular level is summarized in Fig 6.

## Discussion

In the present study, it could be proven that the endocannabinoid system is involved in two different canine inflammatory CNS diseases.

In the last decades, evidence has shown that the functions of the endocannabinoid system are not limited to the CNS but are involved in the whole organism of mammals [42]. Although the endocannabinoid system has been widely studied for its involvement in regulation of neurotransmission [6, 43, 44], increasing evidence supports its involvement in immunomodulation [5, 15, 18, 19, 23, 25] and neuroprotection [45–47]. CB receptors are expressed by leukocytes [14, 20], mice with deficient CB receptors display an altered, usually more severe, inflammatory phenotype [18]. Therefore, the enhancement of the endocannabinoids or the activation of cannabinoid receptors may have valuable therapeutic effects [48].

In accordance with previous studies in other species [39, 49, 50] and in dogs [34], overall CSF levels of AEA were in the picomolar range (pM) and of total AG in nanomolar range (nM). Levels of 2-AG in unstimulated tissues and cells are usually much higher than those of AEA, and are in principle sufficient to permanently activate both cannabinoid receptors [11, 51]. Interestingly, 2-AG spontaneously isomerizes to its biologically inactive form 1-AG, by acyl migration [39]. This phenomenon is of particular importance for quantification of 2-AG. Therefore, both 1-AG and 2-AG were measured and summarized to total AG, since such calculation has been proven to be more accurate [39].

The highest levels of AEA and total AG in CSF were found in dogs with Intraspinal Spirocercosis and differed significantly from all other groups. High values occurred also in SRMA A group that showed significantly higher levels of both AEA and total AG compared to SRMA Tr. Spirocercosis in dogs has been mostly associated with the presence of esophageal granulomas [52], and although the disease is frequently subclinical, esophageal dysphagia is considered the clinical hallmark [52]. Aberrant migration of the worm to unusual anatomical structures results in atypical clinical signs [28]. Neurological deficits presented in the IS group are similar to previous findings of extradural and intraspinal *Spirocerca lupi* migration [30, 53]. Although the reason for aberrant migration is still unclear [28], it possibly occurs via the intercostal arteries, originating from the thoracic aorta, through their spinal branches and into the extradural space [53]. The worm possibly penetrates the dura and pia mater to enter the spinal cord parenchyma [29]. Eosinophils play a major role in dealing with elimination of parasites [54]. CSF samples from the IS group showed mixed neutrophilic pleocytosis with moderate to severe eosinophilia and foamy macrophages. CSF findings agree with previous CSF analysis in single cases of dogs with intraspinal migration [29, 55]. This disease was chosen for the current study, to include a canine population with inflammatory CNS disease and eospinophilia in CSF samples to compare with SRMA dogs and a clear neutrophilic pleocytosis. All CSF samples in the SRMA A group displayed a neutrophilic pleocytosis, the key feature of the acute stage of SRMA [32, 56, 57].

ECs are lipids that are able to modulate cell migration through specific receptors [1]. Both *in vitro* and *in vivo* studies have shown CB2 receptor involvement in eosinophil migration [1], which is supported by the current study. Human peripheral eosinophils express functional CB2 receptors that mediate chemotaxis towards 2-AG, but not AEA [58]. Interestingly, another study showed that 2-AG induces migration of eosinophils in a dose-dependent manner and that the concentration required appears to be pathophysiologically relevant [59]. Since 2-AG is a fully effective CB2 agonist [60], several studies have focused on the immunomodulatory effects of 2-AG, while information of AEA in this aspect is lacking. Moreover, 2-AG has been proven to be produced and released from platelets [61], macrophages [61, 62], endothelial cells [63, 64], glial cells [65], macrophages [62] and adipocytes [66]. The main 2-AG effects on neutrophils seem to be independent of CB activation in humans [67]. Although 2-AG does not seem to have a chemotactive effect on neutrophils, it has been proven to activate them [67].

CSF levels of AEA and 2-AG in healthy dogs were low as already determined in previous measurements [34]. Such low values were also found in dogs under treatment with glucocorticosteroids (SRMA Tr group). Increased levels of AEA have been found in CSF in dogs with epilepsy and correlated with disease severity and duration [34]. AEA seems to regulate seizure threshold in epilepsy [34]. AEA is a high affinity ligand for CB1 receptors [60], mostly expressed in CNS tissue and regulating neurotransmission [68]. AEA CSF levels in SRMA were much higher than in dogs with epilepsy. 2-AG is the fully effective CB2 agonist [60], but AEA has been linked to activate CB2 receptors in pathological conditions [69] and could enhance or regulate the fulminant inflammatory response in SRMA.

AEA concentration in serum was significantly higher in all groups (Intraspinal Spirocercosis, SRMA A, and SRMA Tr) compared to healthy controls. Moreover, dogs suffering from SRMA in the acute phase and those with Intraspinal Spirocercosis showed significantly higher concentrations of total AG than healthy controls in serum. SRMA is a systemic immune-mediated disorder [32]. Even when the main lesions are found in the cervical leptomeninges, SRMA causes systemic inflammatory lesions of the vessels of the heart and intestine [70, 71]. Albeit migration and persistence of larvae or adult worms are responsible for intraspinal lesions in Spirocercosis [72], the tissue damage is not restricted to the spinal cord in these dogs. The systemic nature of both diseases might explain the increased levels of both ECs in serum.

Strong CB2 immunoreaction was found in leukocytes within the marginal zone of splenic follicles and hepatocytes and Kupffer cells as previously described [20, 73, 74]. Previous studies performed in healthy dog tissues have shown strong CB2 immunoreaction in B cell zones of lymphoid follicles, and diffuse immunoreaction in cells in the dermis, including perivascular cells with mast cell morphology, fibroblasts, and endothelial cells [75]. There is however a lack of studies describing CB2 receptor expression in the CNS of dogs. Our study showed moderate to strong CB2 expression on more than 90% ramified glial cells within the grey and white matter of the cervical spinal cord sections of healthy dogs. Moreover, slight to moderate immunoreaction was found in the neurons in the ventral and dorsal horns. CB2 receptor expression, was initially thought to occur only in peripheral tissues (mostly immune organs and cells) [1, 3, 14, 20]. However, several studies have demonstrated CB2 expression in microglia [76–79], oligodendrocytes, astrocytes and endothelial cells in the healthy CNS [80–83]. The expression of CB2 receptors in neurons remains controversial [84]. Nonetheless, functional CB2 receptors have been found in neuronal subpopulations in cerebellum, brainstem, cerebral cortex [85–88], although less concentrated than CB1 receptors [85]. In SRMA and IS CB2 expression seemed to be upregulated both in glial cells and neurons. However, CB2 expression depended on lesion severity and was even downregulated in glial cells in regions with severe necrosis and cell loss. Interestingly, CB2 positive glial cells underwent changes in the morphology in SRMA and IS toward a round morphology showing less and shorter cytoplasmic extensions. Although further studies are needed to exactly confirm the type of glial cells expressing CB2, it might be speculated that the change in morphology is a consequence of astrogliosis and/or correlated with the occurrence of activated microglia. In other studies, upregulation of CB2 receptor expression in microglia, macrophages, neurons and astrocytes upon activation has been demonstrated [5, 86, 89, 90]. An increased production of AEA and 2-AG has also been described in several other pathologies of the CNS [48, 91, 92]. The understanding of the role of AEA in inflammation is particularly important regarding its interaction with microglia [93]. Depending on the relative quantities of 2-AG and AEA that accumulate in certain CNS areas, their competition at CB2 receptors may determine the extent to which CB2 receptors will regulate microglial cell behavior and phenotype, towards a M1 pro-inflammatory phenotype or a M2 anti-inflammatory phenotype [93]. Indeed, CB2 can be induced on demand during early inflammatory events and it has been linked to attenuation of pro-inflammatory cytokine production by microglia, counteracting neuronal damage [1, 42]. On the other hand, upon CB2 activation microglia have also been shown to cause pro-inflammatory actions by eliciting cell migration [65].

CB2 was strongly expressed on infiltrating leukocytes (*i*.*e*. neutrophils, lymphocytes, plasma cells, eosinophils and macrophages) in spinal cord lesions of dogs with SRMA and IS. Interestingly, arachnoid mesothelial-like cells and cells in the pia matter near the lesions expressed also CB2. Immune cells express high levels of CB2, and even a hierarchy of CB2 receptor expression has been described within the immune system (B cells > natural killer cells > monocytes > neutrophils > CD8 lymphocytes > CD4 lymphocytes) [14]. The level of expression, however, is dependent on the activation state of the cell and the type of stimuli [20]. CB2 receptor expression in macrophages has been proven to be modulated upon activation. CB2 is detected at low levels in resting cells, while it is present at high levels in activated macrophages [5, 89].

Endocannabinoids have been shown to downregulate inflammation in numerous experimental models [20–26]. Activation of the cannabinoid system has been linked to decreased inflammatory cell recruitment and enhanced anti-inflammatory cytokine production [18]. Evidence shows that exogenous application of AEA and 2-AG exerts anti-inflammatory effects by decreasing the production of inflammatory mediators [19]. Interestingly, increased levels of AEA and 2-AG have been found in the spinal cord of rats after moderate-severe spinal cord injury (SCI) [48, 94]. The rapid activation of cannabinoid receptors by endocannabinoids after SCI is thought to be an endogenous protective response [48]. In another study, Arevalo-Martin and colleagues showed that one single injection of 2-AG 30 minutes after the induction of the lesion, protects the white matter from secondary damage, reducing the lesion expansion and myelin loss [46], while CB1 and CB2 antagonists increased myelin damage [48]. Strikingly, administration of CB1 and CB2 antagonists impaired the spontaneous motor recovery observed in rats after SCI [48].

Although several studies support endocannabinoids as anti-inflammatory mediators, several *in vitro* and *in vivo* experiments have reported a pro-inflammatory role of the endocannabinoid system in the development of inflammation [18]. These pro-inflammatory effects have been associated with enhanced leukocyte recruitment and activation, production of reactive oxygen species and release of pro-inflammatory cytokines [1, 58, 59, 65, 95, 96]. Nonetheless, most of these pro-inflammatory effects attributed to endocannabinoids involve 2-AG, but not AEA [18]. Indeed, AEA is a potent anti-inflammatory endocannabinoid and acts practically on all cell subsets except natural killer cells and B cells, while 2-AG exerts both pro- and anti-inflammatory effects which seem to be strictly dependent on cell type [97]. The anti-inflammatory effects of AEA may explain the fact that in SRMA Tr AEA serum levels remained high, while the pro-inflammatory total AG levels decreased notably in serum in the SRMA Tr group.

The endocannabinoid system response in inflammation is indeed dependent on type and state of the disease. While CB2 receptor activation in general mediates immunosuppressive effects limiting inflammation and being associated with tissue damage in several pathological conditions, in some disease states activation of the CB2 receptor may enhance or even trigger tissue damage [84]. In physiological conditions, the endocannabinoid production by neurons is high and in microglia low [98]. In diseased CNS tissue with an activated immune system the cell-specific expression profile of cannabinoid receptors changes, resulting in higher expression of CB2 receptors in activated microglia [16, 98], which can secondarily activate astrocytes leading to further induction of expression of inflammatory factors [5]. Such an upregulation on activated microglia cells was also seen in the evaluated canine inflammatory CNS diseases and could be the reason for the excessive immune response in SRMA. As the disorder progresses, the blood brain barrier (BBB) becomes partially disrupted and blood macrophages, B cells, T cells, natural killer cells and other infiltrating leukocytes start upregulating CB2 [5, 42, 93]. The activation of the receptors further stimulates the migration and/or activation of these cells into the nervous tissue and towards the endocannabinoids produced by microglia, neurons [42] and leukocytes [61, 62, 98], which might explain eosinophilia in CSF samples of IS dogs in the current study after extremely high endocannabinoid levels.

Although the initial enhancement of the endocannabinoid system has been proven to have an endogenous protective response and attempts to control inflammation, as the disease progresses, a later dysregulation of the endocannabinoid system during neuroinflammation leads to increased CB2 receptor expression, excessive endocannabinoid production leading to an exacerbated inflammatory response. Thus, both CB2 agonists and antagonists might be beneficial in counteracting the inflammatory consequences depending on the disease phase [42]. Increased AEA, total AG and upregulation of CB2 receptors might therefore explain the waxing and waning course of SRMA.

## Conclusions

The present study revealed an upregulated endocannabinoid system in canines with inflammatory CNS diseases, highlighting the endocannabinoid system as a potential target for treatment of inflammatory CNS diseases. Levels of ECs in CSF of dogs with inflammatory CNS diseases were increased compared with healthy dogs, and those under treatment. Furthermore, dogs with eosinophilic pleocytosis showed higher level of ECs than those with neutrophilic pleocytosis. Such pleocytosis could be due to the effects of ECs on eosinophil migration to the CSF. We also found upregulated expression of CB2 receptors in SRMA and IS lesions. Such an upregulation of AEA and 2-AG together with an overexpression of CB2 receptors may indicate a dysregulation of the endocannabinoid system and may be involved in the inflammatory response. Therefore, the development of new anti-inflammatory treatment strategies in canine CNS inflammation should involve the EC system.
